# Radiation Exposure and Contrast Agent Use during Endovascular Aortic Repair Using Mobile Versus Fixed Angiography Systems

**DOI:** 10.3390/jcdd11030083

**Published:** 2024-02-29

**Authors:** Amir Arnautovic, Waseem Garabet, Reinhold Thomas Ziegler, Joscha Mulorz, Sönke Maximilian Braß, Alexander Oberhuber, Hubert Schelzig, Markus Udo Wagenhäuser, Philip Dueppers

**Affiliations:** 1 Clinic for Vascular and Endovascular Surgery, Department of Vascular and Endovascular Surgery, Medical Faculty and University Hospital Düsseldorf, Moorenstrasse 5, 40225 Düsseldorf, Germany; 2 Clinic for Vascular and Endovascular Surgery, Department of Vascular and Endovascular Surgery, University Hospital Münster, Albert-Schweitzer-Campus 1, 48149 Münster, Germany; 3 Clinic for Vascular Surgery, Department of Vascular Surgery, University Hospital Zurich, Rämistrasse 100, 8091 Zurich, Switzerland

**Keywords:** radiation, contrast media, TEVAR, EVAR, angiography, hybrid operating room, aortic aneurysm

## Abstract

Background: For (thoracic) endovascular aortic repair ((T)EVAR) procedures, both mobile (standard operating room (SOR)) and fixed C-arm (hybrid operating room (HOR)) systems are available. This study evaluated differences in key procedural parameters, and procedural success for (T)EVAR in the SOR versus the HOR. Methods: All patients who underwent standard elective (T)EVAR at the Clinic for Vascular and Endovascular Surgery at the University Hospital Duesseldorf, Germany, between 1 January 2012 and 1 January 2019 were included. Data were retrieved from archived medical records. Endpoints were analyzed for SOR versus HOR during (T)EVAR. Results: A total of 93 patients, including 50 EVAR (SOR (*n* = 20); HOR (*n* = 30)) and 43 TEVAR (SOR (*n* = 22); HOR (*n*= 21)) were included. The dose area product (DAP) for EVAR and TEVAR was lower in the SOR than in the HOR (EVAR, SOR: 1635 ± 1088 cGy·cm^2^; EVAR, HOR: 7819 ± 8928 cGy·cm^2^; TEVAR, SOR: 8963 ± 34,458 cGy·cm^2^; TEVAR, HOR: 14,591 ± 11,584 cGy·cm^2^ (*p* < 0.05)). Procedural fluoroscopy time was shorter in the SOR than in the HOR for EVAR and TEVAR (EVAR, SOR: 7 ± 4 min; EVAR, HOR: 18.8 ± 11.3 min; TEVAR, SOR: 6.6 ± 9.6 min; TEVAR, HOR: 13.9 ± 11.8 min (*p* < 0.05)). Higher volumes of contrast agent were applied during EVAR and TEVAR in the SOR than in the HOR (EVAR, SOR: 57.5 ± 20 mL; EVAR: HOR: 33.3 ± 5 mL (*p* < 0.05); TEVAR; SOR: 71.5 ± 53.4 mL, TEVAR, HOR: 48.2 ± 27.5 mL (*p* ≥ 0.05). Conclusion: The use of a fixed C-arm angiography system in the HOR results in higher radiation exposure and longer fluoroscopy times but lower contrast agent volumes when compared with mobile C-arm systems in the SOR. Because stochastic radiation sequelae are more likely to be tolerated in an older patient population and, in addition, there is a higher incidence of CKD in this patient population, allocation of patients to the HOR for standard (T)EVAR seems particularly advisable based on our results.

## 1. Introduction

Within recent decades, remarkable technical advances in the endovascular field of vascular surgery have enabled surgeons worldwide to provide modern therapeutical options in various vascular beds [1,2]. In this regard, (thoracic) endovascular aortic repair ((T)EVAR) has become the first-line approach for the majority of aortic pathologies, while patients with an extended life expectancy continue to derive significant benefits from open aortic repair [3]. Despite advances, exposing patients to X-rays and contrast agents potentially increases long-term cancer risks and may impair kidney function [4,5]. Surgeons have accepted these adverse effects following a stringent risk-benefit evaluation, as rupture of an abdominal aortic aneurysm (AAA) is lethal in most cases [6]. Nevertheless, reducing radiation exposure and the volume of the applied contrast agent to decrease their respective toxicities is of the highest priority for endovascular therapists [7,8,9].

As a result of the intraoperative angiography and administration of contrast agents, acute kidney injury is one of the most common complications following endovascular aortic therapy, particularly in patients with pre-existing chronic kidney disease. In a study by Brulotte et al. 2013, post-operative contrast-induced nephropathy was described in 2.9% of patients following EVAR [10]. The complexity of endovascular procedures appears to influence the risk of post-operative contrast-induced nephropathy. Here, endovascular branched or fenestrated aortic stent graft placement found such complication in 26% and 29% of analyzed cases, respectively [11]. Deterioration in renal function is generally reversible, but severe forms may occur in rare cases, necessitating temporary or even lifelong dialysis.

For the elective treatment of aortic pathologies using (T)EVAR both mobile C-arms in the standard operating room (SOR) and fixed C-arm angiography systems in the hybrid operating room (HOR) are available. Debates have persisted regarding whether fixed C-arm systems offer lower radiation and contrast agent doses with superior image quality, although some studies have indicated that mobile C-arms significantly reduce radiation exposure in EVAR [12,13,14,15].

Adhering to existing guidelines has been proven to reduce radiation exposure to patients and staff effectively [16,17,18]. In addition, hardware-based supplemental strategies can reduce radiation exposure even further while simultaneously improving imaging quality in the HOR [14]. Similar concepts addressing the same issue have also been introduced in the SOR, suggesting that performing EVAR from an ionizing radiation perspective could be a safer method [13,15]. In addition, alternative contrast agents, such as carbon dioxide (CO_2_) and gadolinium, have also been introduced; however, these agents remain limited to specialized centers and selective cases [16,19].

Given the points mentioned above, it is imperative to thoroughly comprehend the potential risks and benefits associated with the growing population of endovascular aortic patients undergoing treatment in the HOR. Therefore, this retrospective single-center analysis focuses on discerning variances in radiation exposure and contrasting agent volumes administered in the HOR versus the SOR during standard elective (T)EVAR procedures, to effectively minimize these risks by strategically selecting between the two operating room configurations.

## 2. Materials and Methods

Patients who underwent standard elective (T)EVAR for aortic pathologies at the Clinic for Vascular and Endovascular Surgery at the University Hospital Duesseldorf, Germany, between 1 January 2012 and 1 January 2019 were included. Relevant clinical and procedural data were retrieved from archived medical records. All patients received computer-assisted tomography (CAT) scans prior to the procedure. Exclusion criteria were as follows: EVAR or TEVAR for ruptured aneurysms, fenestrated EVAR (fEVAR) as a combined procedure, aorto-monoiliac stent graft placement, and combined EVAR procedures involving the hypogastric artery. Inclusion criteria were as follows: primary EVAR without the necessity for adjunct procedures on the aorta and/or iliac arteries, post-fenestrated stent graft placement EVAR as an individual procedure, and primary TEVAR. For hypothesis-relevant analysis, the patient cohort was divided into two groups based on either the performed procedure (EVAR vs. TEVAR) or the applied angiography system (SOR vs. HOR).

**Figure 1:**
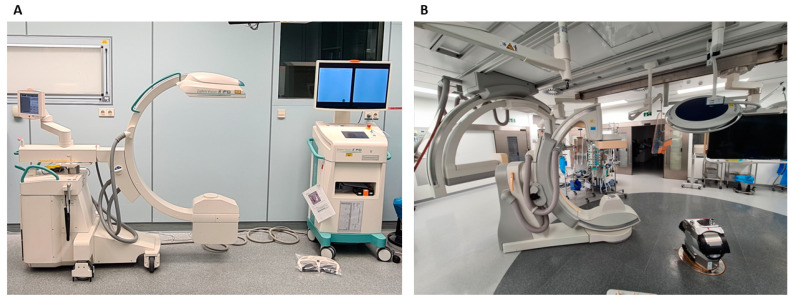
Angiography systems in the Standard Operating Room (SOR) and the Hybrid Operating Room (HOR). (**A**) Exemplary mobile C-arm angiography system from Ziehm Imaging (Ziehm Vision R FD, Nuremberg, Germany) which was used in the SOR. Another similar mobile C-arm angiography system from GE-HEALTHCARE (OEC 9900 Elite, Chicago, IL, USA) as used accordingly. (**B**) Exemplary fixed C-arm angiography system from Philips (Allura Clarity, Amsterdam, Netherlands) which was used in the HOR.

The endpoints of the study were dose area product (DAP), fluoroscopy time, and volume of administered contrast agent. (T)EVAR was performed either in the SOR using mobile C-arms (Ziehm Vision R FD, Nuremberg, Germany; OEC 9900 Elite, GE-HEALTHCARE, Chicago, IL, USA) (Figure 1A) or in the HOR using a fixed angiography system (Philips AlluraClarity, Amsterdam, Netherlands) (Figure 1B). The settings of the C-arms were as follows: for the SOR, 12 frames per second (fps), voltage 45 kV, and radiation energy 10 mAs; for the HOR, 2 FPS, voltage 80 kV, and radiation energy 7 mAs. All procedures were performed by experienced endovascular surgeons. The absence of type I and type III endoleaks (EL) on the final angiogram was defined as procedural success.

Categorical variables are presented as frequency distribution and percentage, and continuous variables with mean ± standard deviation (SD). All variables were tested for normal distribution using the Shapiro–Wilk test. For non-normally distributed parameters, a Chi-Square test was used to compare frequency distributions between two categorical variables. The Mann–Whitney-U test and the Wilcoxon rank-sum test were used to compare rank sums/mean ranks between two independent/different groups. A *p*-value of <0.05 was considered statistically significant. The institutional ethics committee granted approval for this study (2019-402-RetroDEua).

## 3. Results

The patient cohort consisted of 93 patients, including 25 females and 68 males, at a mean age of 71.8 ± 7.7 years. In total, 50 EVAR and 43 TEVAR procedures were performed. The patient cohorts were similar with regard to patient characteristics and key morphological data for the SOR and HOR cohorts for both EVAR and TEVAR procedures (Table 1 and Table 2).

Radiation exposure was less for the SOR group than for the HOR group. In detail, during EVAR procedures, the DAP was 1.6 × 10^3^ ± 1.1 × 10^3^ cGy·cm^2^ for the SOR cohort and 7.8 × 10^3^ ± 8.9 × 10^3^ cGy·cm^2^ for the HOR cohort (*p* < 0.001). Similar observations were obtained for TEVAR procedures. In these cases, DAP was 9 × 10^3^ ± 3.4 × 10^4^ for the SOR cohort and 1.5 × 10^4^ ± 1.2 × 10^3^ cGy·cm^2^ for the HOR cohort (*p*< 0.001). In line with this finding, the data also showed that total procedural fluoroscopy time was longer in the HOR when compared to the SOR; this condition was true for both EVAR (SOR: 7.08 ± 4 min; HOR: 18.8 ± 11.4 min) and TEVAR (SOR: 6.6 ± 9.6 min; HOR: 13.9 ± 11.8 min, *p* < 0.001). No differences were found in total procedure time for EVAR or TEVAR in the SOR vs. HOR (Table 3). The volume of administrated contrast agent was lower during EVAR in the HOR when compared to the SOR (SOR = 57.5 ± 20 mL; HOR= 33.32 ± 5 mL) (*p* < 0.001). Similar observations were obtained for TEVAR, but they failed to show statistical significance (SOR: 71.5 ± 53.4 mL; HOR = 48.2 ± 27.5 mL) (*p* = 0.08) (Table 3). Overall, procedural success was 91.4%. Complete exclusion of aneurysms from blood flow was achieved in 100% of all EVAR procedures and 97.7% of all TEVAR procedures, with similar rates in the SOR and the HOR. On final angiograms, we observed eight Els, of which seven were of type II. In detail, there were two type II ELs in the SOR, three type II ELs in the HOR during EVAR, and one type II EL each in both the SOR and HOR during TEVAR. Of note, there was one type I EL in the HOR during TEVAR, which was resolved by proximal stent graft extension (*p* ≥ 0.05). Conversions to open surgery, deaths within 24 h post-surgery, or complete occlusions of the stent graft were not seen in the cohorts of this study (Table 3).

## 4. Discussion

This study found higher radiation exposure, longer fluoroscopy time, and lower contrast agent volume with comparable procedural success rates during standard aortic procedures such as EVAR and TEVAR in the HOR as compared to the SOR. These results add to the experiences in the current literature, which has comprehensively reported congruent findings addressing similar hypotheses in comparable patient cohorts.

To date, several studies have reported on experience with radiation exposure during standard, uncomplicated EVAR procedures. In this regard, Martinez et al. reported a higher radiation exposure during EVAR in the HOR using fixed angiographic equipment compared to the HOR [13]. Similar to that result, Schaefers et al. described in their cohort of 160 patients a lower radiation exposure when using a mobile C-arm angiography system in standard EVAR procedures [15]. Other authors have reported a median DAP of 1.07 × 10^4^ cGy·cm^2^ in 1700 EVAR treatments, while we have reported here a lower overall DAP for both the SOR and the HOR [20]. In contrast to these reports, Rehman et al. reported significantly lower radiation exposure, shorter screening times, and lower use of contrast agent among 286 EVAR patients assessed between 2009 and 2016 during standard EVAR procedures in the HOR versus the SOR, suggesting performance of those standard procedures in a dedicated vascular HOR [21]. Informative data were further reported by Ruiter et al. in their meta-analysis, which summarized a total of 27 studies involving 3444 patients who had received endovascular aortic therapy, including (T)EVAR, fenestrated EVAR (fEVAR) and/or branched EVAR (bEVAR) using either a mobile or a fixed radiation source. The authors found that, for equivalent fluoroscopy times, the use of a fixed C-arm in noncomplex aortic procedures had generated higher patient radiation doses compared to a mobile C-arm. Of note, more complex aortic procedures, such as fenestrated and/or branched stent graft placements, had generated the highest radiation dose per interventions; therefore, the authors recommended applying fusion imaging techniques to compensate for this condition [22]. Comprehensive inter-study comparisons are nearly impossible because no standardized protocol currently exists regarding, for example, contrast agent volume dilution or angiography time for target vessel marking (Table 4).

Because most of the aforementioned studies have suggested lower radiation exposure and fluoroscopy times in the SOR versus the HOR, it seems worthwhile to state here that radiation exposure does not depend exclusively on whether a fixed or mobile angiography system is employed. Although outside the scope of the present study, some data have suggested that a more modern, permanently installed imaging system such as the AlluraClarity (Phillips, Amsterdam, the Netherlands), which was used for the analysis in this study, may produce lower radiation exposure when applying digital subtraction angiography (DSA) as compared to other systems during similar endovascular procedures [14]. Furthermore, radiation exposure may also heavily depend on procedure-specific parameters such as the duration and number of angiographies, the position of the radiation source relative to the patient, the patient’s body mass index, and the distance of the radiation source from the patient [23]. In addition, anatomical landmarks such as neck angulation, AAA diameter, neck diameter, and type of AAA may affect radiation exposure levels [24]. For instance, such hostile anatomical configurations may require longer fluoroscopy times because they are technically more complex; however, such considerations may be challenged by other studies that have reported no difference in radiation exposure during EVAR procedures in patient cohorts with different neck lengths in emergency versus elective settings [13]. In this regard, factors other than anatomical landmarks, such as the choice of stent graft and the application of fusion imaging techniques, may contribute even more to the radiation dose level than the choice of fixed versus mobile angiography systems themselves. All these parameters, however, must be addressed in future studies to optimize procedural protocols and reduce fluoroscopy exposure for both patients and staff.

In addition to EVAR, TEVAR is also performed in an HOR at most vascular centers today. The present study detected lower radiation exposure for TEVAR in the SOR as compared to the HOR, congruent with our findings for EVAR procedures. The literature has provided less evidence for this endpoint, although the above-mentioned meta-analysis found similar results for uncomplicated TEVAR as compared to EVAR in reporting a lower radiation exposure [22].

Another quality indicator in endovascular surgery is the volume of the contrast agent applied during the procedure. Because endovascular procedures are often performed in elderly patients who frequently have impaired kidney function, the judicious use of contrast agents is essential [5]. Notably, our results have clearly demonstrated lower contrast agent use in the HOR versus the SOR during both EVAR and TEVAR procedures. Although this finding would seem understandable as advanced imaging quality may minimize the necessity for contrast agent use, there have been contrary reports regarding the potential benefit of an HOR towards such an endpoint in the literature [13,15,21]. Future studies involving larger patient cohorts are essential to determine whether variations in contrast agent volumes significantly impact rates of kidney injury.

In addition to the radiation exposure of the patient, the radiation exposure of the staff who work in the HOR has also gained interest in recent years. The HOR contains numerous structural solutions that contribute to effective radiation protection, such as under-table radiation protection and a lead glass pane for shielding the upper body. Alongside these structural features, there are also methodological and technical elements that promote radiation protection, including the detector size and the option for road mapping. In addition to these elements, larger room sizes allow working at a greater distance from the radiation source, which is beneficial for the staff [25].

This study found a total of eight ELs on the final angiogram, of which only one type I EL occurred during TEVAR in the HOR. Although more ELs occurred in the HOR when considering both investigated procedures, there was no statistical difference in total EL rates when comparing those procedures between the SOR and the HOR. The most common EL following EVAR is type II, and particular interest has arisen in preventing such retrograde AAA sac perfusion for this reason. Although success rates of >95% without any endoleak have been described and potential threshold values of the number of targeted lumbar arteries have been introduced, scientific debate regarding the effectiveness of this approach is ongoing [26,27]. While early experiences could not identify a direct correlation between the embolization of the inferior mesenteric artery and/or lumbar arteries prior to stent graft placement, more recent reports have observed great potential to reduce EL type II rates [28,29].

This study has some notable limitations. It involved a retrospective, single-center data analysis; thus, the reported results are somewhat difficult to generalize and may possibly be heavily biased. In addition, the patient cohort sizes may have been insufficient for drawing valid conclusions. Because not all factors potentially contributing to DAP and fluoroscopy time were evaluated, substantial confounding may have been possible. Due to the limited sample size of the study, adjusted analysis for differences between the groups could not be conducted. Finally, and importantly, protocols related to applied contrast agent dilutions, for example, may change over time, possibly biasing the reported results. 

## 5. Conclusions

In summary our data advocate that (T)EVAR with mobile C-arm angiography systems still has its place, and can generate comparable results when compared to fixed C-arm angiography systems at lower DAP and fluoroscopy time. Moreover, the adoption of HOR may lower contrast agent volumes, which in turn may potentially decrease the risk of kidney injury. This aspect could be particularly beneficial for elderly patients with pre-existing kidney function impairment. More complex endovascular aortic procedures, however, may benefit from advanced imaging techniques in modern HOR. 

## Figures and Tables

**Table 1:** Patient characteristics and key morphological data. Data are presented as absolute and relative frequencies *n* (%) or mean ± standard deviation for the standard operating room (SOR) and the hybrid operating room (HOR) during standard EVAR procedures. Patient cohorts for the SOR and HOR showed similar characteristics. A Mann–Whitney-U Test or a Chi-square test were applied were applicable. (*n* = 50).

Variable	EVAR (*n* = 50)		
	SOR (*n* = 20)	HOR (*n* = 30)	*p*-Value
Male	18 (90)	24	0.35
Female	2 (10)	6	
Age [y]	73.2 ± 9.6	72.3 ± 10.2	0.90
CHD (*n* = 50)	13 (65)	17 (57)	0.56
T2DM (*n* = 50)	3 (15)	5 (17)	0.88
COPD (*n* = 50)	4 (20)	6 (20)	1
CKI (*n* = 50)	5 (25)	3 (10)	0.16
smoking (*n* = 20)	2 (40)	8 (53)	0.61
HCL (*n* = 48)	10 (50)	10 (35)	0.03
Risk classification			
ASA I	0	2 (7)	
ASA II	3 (15)	7 (23)	
ASA III	14 (70)	14 (47)	0.13
ASA IV	1 (5)	2 (10)	
ASA V	0	0	
AAA	12 (60)	26 (87)	0.12
Juxtarenal AAA	2 (10)	0	
TAAA	1 (5)	1 (3)	
TAA	0	0	
TBAD	0	0	
PAU infrarenal	5 (25)	3	
Aneurysm diameter [mm]	59.2 ± 11.8	53.82 ± 5.26	0.38

CHD: coronary heart disease, T2DM: type 2 diabetes mellitus, COPD: chronic obstructive pulmonary disease, CKI: chronic kidney injury, HCL: hypercholesterinemia, ASA: American Society of Anesthesiologists, AAA: abdominal aortic aneurysm, TAAA: thoraco-abdominal aortic aneurysm, TAA: thoracic aortic aneurysm, TBAD: type B aortic dissection, PAU: penetrating aortic ulcer, EVAR: endovascular aortic repair, SOR: standard operating room, HOR: hybrid operating room.

**Table 2:** Patient characteristics and key morphological data. Data are presented as absolute, and relative frequencies *n* (%) or mean ± standard deviation for the standard operating room (SOR) and the hybrid operating room (HOR) during standard TEVAR procedures. Patient cohorts for the SOR and HOR showed similar characteristics. A Mann–Whitney-U Test or a Chi-square test were applied where applicable. (*n* = 43).

Variable	TEVAR (*n* = 43)		
	SOR (*n* = 22)	HOR (*n* = 21)	*p*-Value
male	15 (68)	11 (52)	0.29
female	7 (32)	10 (48)	
age [y]	68.7 ± 10.3	72.6 ± 8.9	0.31
CHD (*n* = 43)	8 (36)	15 (71)	0.02
T2DM (*n* = 43)	4 (18)	3 (14)	0.73
COPD (*n* = 43)	9 (41)	8 (38)	0.90
CKI (*n* = 43)	6 (27)	9 (43)	0.28
smoking *n* = 19	4 (57)	6 (50)	0.76
HCL (*n* = 43)	6 (27)	9 (43)	0.28
Risk classification			
ASA I	1 (5)	0	
ASA II	2 (9)	2 (10)	
ASA III	14 (64)	15 (71)	0.49
ASA IV	1 (5)	0	
ASA V	0	0	
AAA	0	0	0.83
AAA juxtarenal	1 (5)	0	
TAAA	12 (55)	11 (52)	
TAA	4 (18)	3 (14)	
TBAD	3 (14)	4 (19)	
PAU thoracic	2 (9)	3 (14)	
Aneurysm diameter	62.4 ± 8.9	65.3 ± 14	0.51

CHD: coronary heart disease, T2DM: type 2 diabetes mellitus, COPD: chronic obstructive pulmonary disease, CKI: chronic kidney injury, HCL: hypercholesterinemia, ASA: American Society of Anesthesiologists, AAA: abdominal aortic aneurysm, TAAA: thoraco-abdominal aortic aneurysm, TAA: thoracic aortic aneurysm, TBAD: type B aortic dissection, PAU: penetrating aortic ulcer, EVAR: endovascular aortic repair, SOR: standard operating room, HOR: hybrid operating room.

**Table 3:** Procedural data and endoleak (EL) rates. Data are presented as absolute and relative frequencies *n* (%) or mean ± standard deviation for the standard operating room (SOR) and the hybrid operating room (HOR) during standard EVAR and TEVAR procedures. A Mann–Whitney-U Test or a Chi-square test were applied were applicable. (*n* = 93).

Variable	EVAR (*n* = 50)			TEVAR (*n* = 43)		
	SOR (*n* = 20)	HOR (*n* = 30)	*p*-Value	SOR (*n* = 22)	HOR (*n* = 21)	*p*-Value
DAP (cGy·cm^2^)	1.64 × 10 × 10^3^ ± 1.09 × 10 × 10^3^	7.8 × 10 × 10^3^ ± 8.9 × 10 × 10^3^	<0.001	8.96 × 10 × 10^3^ ± 3.45 × 10 × 10^4^	1.46 × 10 × 10^3^ ± 1.16 × 10 × 10^4^	<0.001
Fluoroscopy time (min)	7.1 ± 4	18.4 ± 11.4	<0.001	6.6 ± 9.6	13.90 ± 11.8	<0.001
Procedural time (min)	127.7 ± 37.3	127.5 ± 36.2	1	118.1 ± 47.1	118.00 ± 60.7	0.79
Contrast agent (mL)	57.4 ± 23.6	33.3 ± 24.6	<0.001	71.5 ± 53.4	48.2 ± 27.5	0.08
No EL	18 (90)	27 (90)	1	21 (96)	19 (86)	0.59
Type I EL	0	0		0	1 (5)	
Type II EL	2 (10)	3 (10)		1 (5)	1 (5)	

DAP: dose area product, EL: endoleak, SOR: standard operating room, HOR: hybrid operating room, EVAR: endovascular aortic repair, TEVAR: thoracic endovascular aortic repair.

**Table 4:** Literature review of dose are products in the standard (SOR) and hybrid operating room (HOR). The table summarizes recent results in EVAR procedures with reported dose are product (DAP) [cGy·cm^2^] in the SOR and HOR.

Study	EVAR		
	SOR	HOR	*p*-Value
Present study (cGy·cm^2^)	1.64 × 10 × 10^3^ ± 1.19 × 10 × 10^3^	7.82 × 10 × 10^3^ ± 8.93 × 10 × 10^3^	<0.001
Martinez et al. 2020 (cGy·cm^2^) [13]	6.15 × 10 × 10^3^ ± 4.24 × 10 × 10^3^	1.54 × 10 × 10^4^ ± 1.03 × 10 × 10^4^	<0.005
Schaefers et al. 2018 (cGy·cm^2^) [15]	4.99 × 10 × 10^3^ ± 3.81 × 10 × 10^3^	1.68 × 10 × 10^4^ ± 1.47 × 10 × 10^4^	<0.001
Rehman et al. 2019 (cGy·cm^2^) [21]	1.68 × 10 × 10^4^	8.23 × 10 × 10^3^	<0.001

## Data Availability

The underlying data are available from the corresponding author upon reasonable request.
